# Feeding practices and risk factors for chronic infant undernutrition among refugees and migrants along the Thailand-Myanmar border: a mixed-methods study

**DOI:** 10.1186/s12889-019-7825-7

**Published:** 2019-11-28

**Authors:** A. H. Hashmi, P. B. Nyein, K. Pilaseng, M. K. Paw, M. C. Darakamon, A. M. Min, P. Charunwatthana, F. Nosten, R. McGready, V. I. Carrara

**Affiliations:** 1 Shoklo Malaria Research Unit, Mahidol-Oxford Tropical Medicine Research Unit, Faculty of Tropical Medicine, Mahidol University, Mae Sot, 63110 Thailand; 2 Department of Family Medicine, Faculty of Medicine, Chiang Mai University, Chiang Mai, 50200 Thailand; 3 Mahidol-Oxford Tropical Medicine Research Unit, Faculty of Tropical Medicine, Mahidol University, Bangkok, 10400 Thailand; 4 Centre for Tropical Medicine and Global Health, Nuffield Department of Medicine, University of Oxford, Oxford, OX37FZ UK; 5 Department of Medicine, Swiss Tropical and Public Health Institute, Socinstrasse 57, CH-4051 Basel, Switzerland

**Keywords:** Infant feeding behaviour, Complementary feeding, Infant and child nutrition, Nutrition education, Infant Nutritional Physiological Phenomena, Refugees, Transients and migrants

## Abstract

**Background:**

This study aims to provide a comprehensive understanding of maternal risk factors, infant risk factors and maternal infant feeding practices among refugees and migrants along the Thailand-Myanmar border.

**Methods:**

This study employed a mixed-methods approach with two components: (1) cross-sectional survey (*n* = 390) and (2) focus group discussions (*n* = 63). Participants were chosen from one of three clinics providing antenatal and delivery services for Karen and Burman refugees and migrants along the border. Participants were pregnant women and mother-infant dyads.

**Results:**

Refugee and migrant mothers demonstrated high rates of suboptimal breastfeeding and low rates of minimum dietary diversity and acceptable diet. Multivariable regression models showed infant stunting (AOR: 2.08, 95% CI: 1.12, 3.84, *p* = 0.020) and underweight (AOR: 2.26, 95% CI: 1.17, 4.36, *p* = 0.015) to have increased odds among migrants, while each 5 cm increase in maternal height had decreased odds of stunting (AOR: 0.50, 95% CI: 0.38, 0.66, *p* < 0.001) and underweight (AOR: 0.64, 95% CI: 0.48, 0.85, *p* = 0.002). In addition, small-for-gestational-age adjusted for length of gestation, infant age and gender increased odds of infant’s stunting (AOR: 3.42, 95% CI: 1.88, 6.22, p < 0.001) and underweight (AOR: 4.44, 95% CI: 2.36, 8.34, *p* < 0.001). Using the Integrated Behavioural Model, focus group discussions explained the cross-sectional findings in characterising attitudes, perceived norms, and personal agency as they relate to maternal nutrition, infant malnutrition, and infant feeding practices.

**Conclusions:**

Inadequate infant feeding practices are widespread in refugee and migrant communities along the Thailand-Myanmar border. Risk factors particular to maternal nutrition and infant birth should be considered for future programming to reduce the burden of chronic malnutrition in infants.

## Background

Infant undernutrition and growth failure during the first 1000 days of life can have deleterious long-term effects extending through adolescence and into adulthood [1–4]. In 2011, it was estimated that undernutrition, including fetal growth restriction, stunting, wasting and suboptimal breastfeeding accounted for 45% of all child deaths [5]. Low- and middle-income countries (LMIC) bear the greatest burden of chronic undernutrition as determined by stunting and underweight in children. Asia has some of the highest rates, with at least 25% of children under 5 years of age stunted and approximately 20% underweight [5].

Recent analyses reveal that growth faltering can be detected in early infancy [6–8], when suboptimal breastfeeding and inadequate complementary feeding become strong risks for chronic undernutrition in infants [9]. A recent cohort study across 8 LMIC demonstrated high rates of suboptimal breastfeeding, with 6 of 8 sites reporting < 60% exclusively breastfed and > 20% partially breastfed infants at 1 month of life [10].

Of particular interest are refugees and migrants residing along the Thailand-Myanmar border who are at increased risk of infant and child malnutrition [11]. Among refugees residing in Thailand, poor infant growth has been documented with rates of stunting reaching 18% from 6 to 11 months and increasing to 34% by the second year of life [12]. Estimates of exclusive breastfeeding in Myanmar—where Karen and Burman refugees and migrants residing in Thailand predominantly originate from—range from 24 to 41% in a cross-sectional analysis, while adequate feeding from birth to 11 months range from 41 to 63% [13–15].

The literature has demonstrated links between maternal and infant risk factors and chronic undernutrition. Maternal under-nutrition, potentially exacerbated by suboptimal gestational weight gain, can lead to poor pregnancy outcomes that can influence an infants’ risk for stunting and underweight. Under-nutrition in pregnancy is related to pre-eclampsia [16], fetal growth restriction, low birth weight [17–19] and small-for-gestational-age [16, 18, 20, 21] and impacts neonatal and infant mortality and morbidity. Maternal anthropometry, such as low maternal BMI and short stature (height < 145 cm) put infants at higher risk for preterm birth and small-for-gestational-age [5, 22–24]. Furthermore, contextual factors such as limited parental educational attainment, poverty and fewer antenatal and postnatal visits are associated with inadequate feeding practices and chronic malnutrition in infants in Asia [25–31]. Specific to the refugees and migrants along the border, low maternal literacy rates [32]; unstable income and unemployment; and limited access to quality food and food resources [11, 32, 33] can play a role in child undernutrition. By contrast, consumption of solid foods, a minimum acceptable diet and dietary diversity between 6 and 8 months significantly reduced the risk of stunting and underweight in 14 LMIC [34].

Although high rates of breastfeeding initiation and appropriate breastfeeding duration nearing 2 years have been documented for refugee and migrant communities along the Thailand-Myanmar border [35], breastfeeding and complementary feeding practices in infancy as risk factors for chronic undernutrition in this population remain poorly understood. There are limited data on maternal and contextual risk factors that could also influence the high rates of chronic undernutrition observed in these populations. Therefore, this study employs a mixed-methods approach that aims to provide a comprehensive understanding by evaluating maternal risk factors, infant risk factors and maternal infant feeding practices as they relate to chronic undernutrition among Karen and Burman refugee and migrant infants.

## Methods

### Study setting and population

**Fig. 1:**
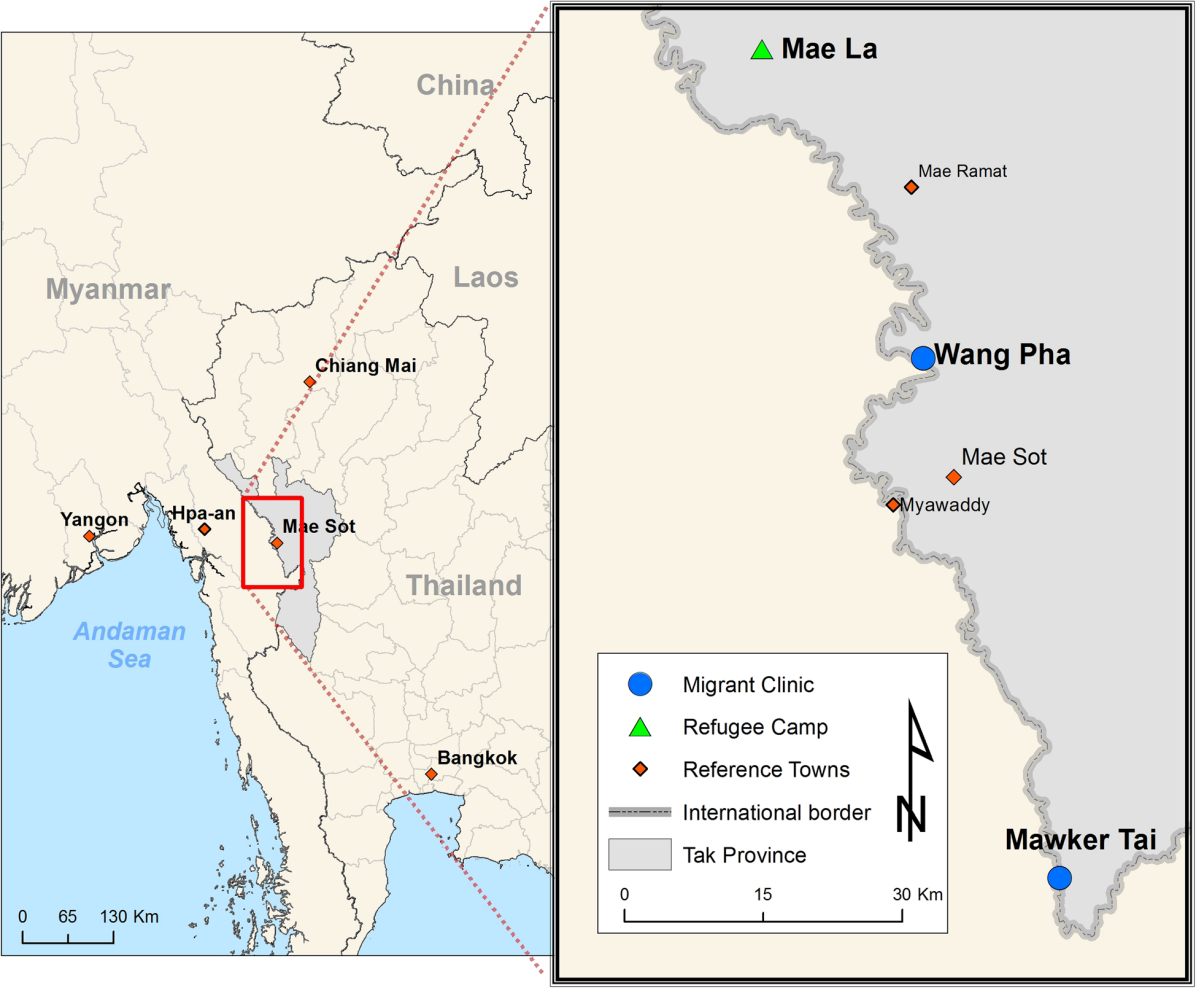
Map of Thailand-Myanmar border showing SMRU clinic sites in Mae La refugee camp, Mawker Thai and Wang Pha. (*Credit to Daniel Parker, former researcher at SMRU, with permission to use for this publication*)

This study was conducted at the Shoklo Malaria Research Unit (SMRU) with refugees in Mae La camp (MLA), and migrants in Mawker Thai (MKT) and Wang Pha (WPA) communities, along the Thailand-Myanmar border (Fig. 1). SMRU clinics provide antenatal (ANC), obstetric, paediatric and general medical care. SMRU is currently headquartered in Mae Sot, Thailand, but began providing health services and performing health research in the Shoklo refugee camp in 1986. Following closure of Shoklo refugee camp in 1998, SMRU moved operations to serve refugees resettled in MLA. As Thailand witnessed a rise in migrant populations through the 1990s, often suffering from malaria, SMRU established MKT and WPA clinics in 1998 and 2004, respectively [36]. In 2014, The Border Consortium estimated a total of 114,569 refugees receiving food rations in the camps within Thailand, with MLA housing the largest number of refugees at 42,542 individuals [37]. SMRU’s migrant clinics (MKT, WPA) estimate a catchment of nearly 200,000 individuals [38].

Refugees and migrants are predominantly of Burman or Karen ethnicity who identify themselves as Buddhist or Christian. Muslims constitute a notable minority within the MLA refugee camp.

### Study subjects

Subjects were selected from a cohort evaluating the impact of malaria in pregnancy on fetal growth. Mother-infant dyads with infants aged 2 to 12 months at the time of the survey participated once in the cross-sectional survey and women still pregnant enrolled in the focus group discussions (FGD). All participants were invited to join the study as they reported for regular follow-up at SMRU clinics.

### Study design

The mixed-methods approach utilised a sequential explanatory design with the cross-sectional survey (March–May 2014) performed prior to FGD (May-Jun 2014). This allowed for more robust characterization of maternal infant feeding practices: where cross-sectional survey provided static indicators of infant feeding and water, sanitation and hygiene (WASH) behaviours, FGD provided more of a “narrative” to elicit a deeper understanding of attitudes underlying the infant feeding practices observed.

The cross-sectional survey and FGD guide were originally drafted in English, translated into Karen and Burmese, then back-translated, and further corrected after piloting with pregnant women and mothers in Karen and Burmese, before being finalised for use.

### Cross-sectional survey

Mothers with infants aged 2 to 12 months were provided information about the study, and once consent was given, responded to a one-time, staff-administered 25-question survey which included WHO recommendations for appropriate infant feeding [39]; socioeconomic indicators; household expenditures for food and access to food resources; and access to and type of sanitation used by the household following definitions from the UNICEF Thailand Multiple Indicator Cluster Survey [40]. Food resources were categorized as: refugee rations; markets; shops (smaller in size and diversity of foods compared to markets); livestock or vegetables grown and used for food at home; and food gathered from fields or forests near the home.

### Focus group discussions

FGD with pregnant women were designed as “elicitation discussions” as prescribed by the Integrated Behavioural Model (IBM)—a model that rests on the assumption that the most important determinant of an individual performing a behaviour is their intention to perform said behaviour [41, 42]. FGD guides were developed specifically for this study and focused on eliciting participant attitudes; perceived cultural norms and normative influences on behaviours; and personal agency related to: infant malnutrition; infant feeding practices (exclusive breastfeeding, complementary feeding, dietary diversity, and meal amounts and frequency); and maternal malnutrition and overnutrition (Additional file 1). Definitions for attitudes, perceived norms and personal agency followed the framework provided by the IBM. Attitudes considered two facets: experiential (positive or negative feelings about performing a particular behaviour) and instrumental (positive or negative outcomes of performing a given behaviour). An important element of perceived norms that were specifically sought in FGD was the normative influences on behaviours—that is, individuals or groups within a participant’s social environment that may support or oppose a given behaviour. This also included some elements of a participant’s motivation to comply with such advice. Given this low resource setting, the authors found particular utility in the IBM conceptualisation of “personal agency” that encompasses both perceived control (facilitators or barriers to perform a given behaviour) and self-efficacy (a participant’s belief or certainty that given potential barriers, one could perform a given behaviour). As an important element of self-efficacy, the knowledge required to implement an action one deems appropriate was also considered under this construct [42, 43].

FGD were led by a native Karen, fluent in Sgaw and Pwo Karen, Burmese and English and trained in this methodology [44]. Purposive sampling was attempted to create groups of 4–8 participants similar in ethnicity, language, parity and age to allow for deeper discussion.

### Variables of interest

#### Maternal anthropometry, malaria, anaemia, and demographics

Trained midwives measured weight upon first ANC consultation and at each follow-up visit using medical-grade, mechanical scales with 0.5 kg precision. Maternal weight at delivery was defined as the last weight taken between 34 + 0 and 41 + 6 weeks of gestation. Gestational weight gain was the difference between delivery weight and weight at first ANC consultation for women with a first trimester (< 14 weeks’ gestation) weight measurement, categorised as < 10 kg, 10–15 kg or ≥ 15 kg [16]. Height at first ANC was measured by stadiometers allowing for precision up to 1 mm. Body mass index (BMI) was measured from weight and height determined at first ANC visit for women reporting in the first trimester, and categorised as outlined by the WHO expert consultation on Asian populations: low (BMI < 18.5 kg/m^2^), normal (BMI 18.5–22.9 kg/m^2^) and high (BMI ≥ 23 kg/m^2^) [45].

Available demographic variables collected at first ANC consultation were: maternal age, ethnicity (Burman, Karen or other), religious preference (Buddhist, Christian, Muslim or other), length of residency at current address, parity and refugee or migrant status. Women self-reported literacy as “able to read” and “able to write.” Smoking status was self-reported as a dichotomous variable. At first ANC and subsequent visits, malaria infection (any species) was determined by microscopy of peripheral blood smear. Haematocrit is collected routinely at first ANC visit, with haematocrit less than 30% used to diagnose anaemia. Total number of ANC visits were recorded over the course of gestation until delivery and categorised as ≥ 8 or < 8 visits [46]. Additional demographic data collected as part of the cross-sectional survey included: paternal education in years, weekly food expenditure per household member; the number of food resources that the mother could access (rations, market, shops, cultivated at home, or picked from neighbouring forests); access to sanitation categorised as improved (protected pit latrine or pour-flush/flush toilet) and unimproved (unprotected latrines or open defecation); and distance travelled to a SMRU clinic (< 30 min vs ≥ 30 min).

#### Infant variables: birth weight, birth length, preterm birth, and small-for-gestational-age

Gestational age was calculated from the mean of two first trimester crown-rump-length measurements at first ANC (before gestational age of 14 + 0 weeks) using an established formula [47] or the mean of two biparietal diameter measurements (before 24 + 0 weeks) using an Asian formula [48]. Preterm birth was defined as birth before 37 weeks’ gestation. Staff were trained to take all infant anthropometric measurements with quality control in place to monitor measurement accuracy and precision. Birth length and length at time of cross-sectional survey was taken using the UNICEF infant height/length measuring board (precision to 1 mm). Birth weight was recorded using Seca electronic scales (precision to 5 g), subsequent weight was measured with Seca mechanical scales (precision 50 g). At birth, infants were determined as small-for-gestational-age if birth weight was at the 10th centile or below for appropriate gestational age [49]. Infant age at time of cross-sectional survey was also recorded.

#### Maternal infant feeding and water, sanitation and hygiene (WASH) practices

Table 1 defines infant feeding practices studied in the cross-sectional survey based on WHO indicators for appropriate feeding: exclusive breastfeeding, minimum dietary diversity, meal frequency and minimum acceptable diet [39], with additional hygiene and sanitation behaviours included.

**Table 1:** Outcomes of interest for appropriate feeding, water, hygiene and sanitation practices in the 24 h prior to cross-sectional survey [39]

Outcomes of interest	Definition
Exclusive breastfeeding	Infants < 6 months fed breast milk while receiving no other food or liquid, not even water, except for vitamins, mineral supplements, or medicine
Predominant breastfeeding	Infants < 6 months receiving predominantly breast milk and also receiving liquids, but not soft food
Partial breastfeeding	Infants < 6 months receiving breast milk and any soft food
Dietary diversity	Infant ≥ 6 months fed 4 or more food groups
Meal frequency	Minimum of 2 meals for infants 6–8 months and 3 meals for infants 9–12 months if breastfed; minimum of 4 meals aged 6–12 months if not breastfed
Minimum acceptable diet	Infant ≥ 6 months meeting minimum requirements for dietary diversity and meal frequency
Handwashing	Of mothers preparing meals, those who appropriately washed hands before meal preparation
Safe stool disposal	Of mothers with an improved form of sanitation (latrine, flush or pour-flush toilet), those that discarded infant stool using an improved form of sanitation
Safe water	Infants ≥ 6 months fed boiled or bottled water

### Outcomes of interest: infant stunting and underweight

Stunting and underweight were both determined by the length- and weight-for-age of the infant at the time of cross-sectional survey and were defined as height-for-age < − 2 Z-score and weight-for-age < − 2 Z-score, respectively, using WHO guidelines [50].

### Statistical analysis

Cross-sectional survey data were analysed using Stata version 14 (StataCorp LP, College Station, TX, USA). Continuous data were described by their mean and standard deviation (SD) or their median and range and compared with the independent Student’s t-test or the Mann-Whitney U test depending on the normality of variable distribution. Proportions were used to describe categorical data which were compared using the Chi-squared test with Yates’ continuity correction, the Fisher’s exact test, or an appropriate test for trend for comparisons across sites. Collinearity between demographic variables was tested using Spearman’s rank correlation coefficient or Pearson’s correlation coefficient for categorical and continuous variables, respectively.

**Fig. 2:**
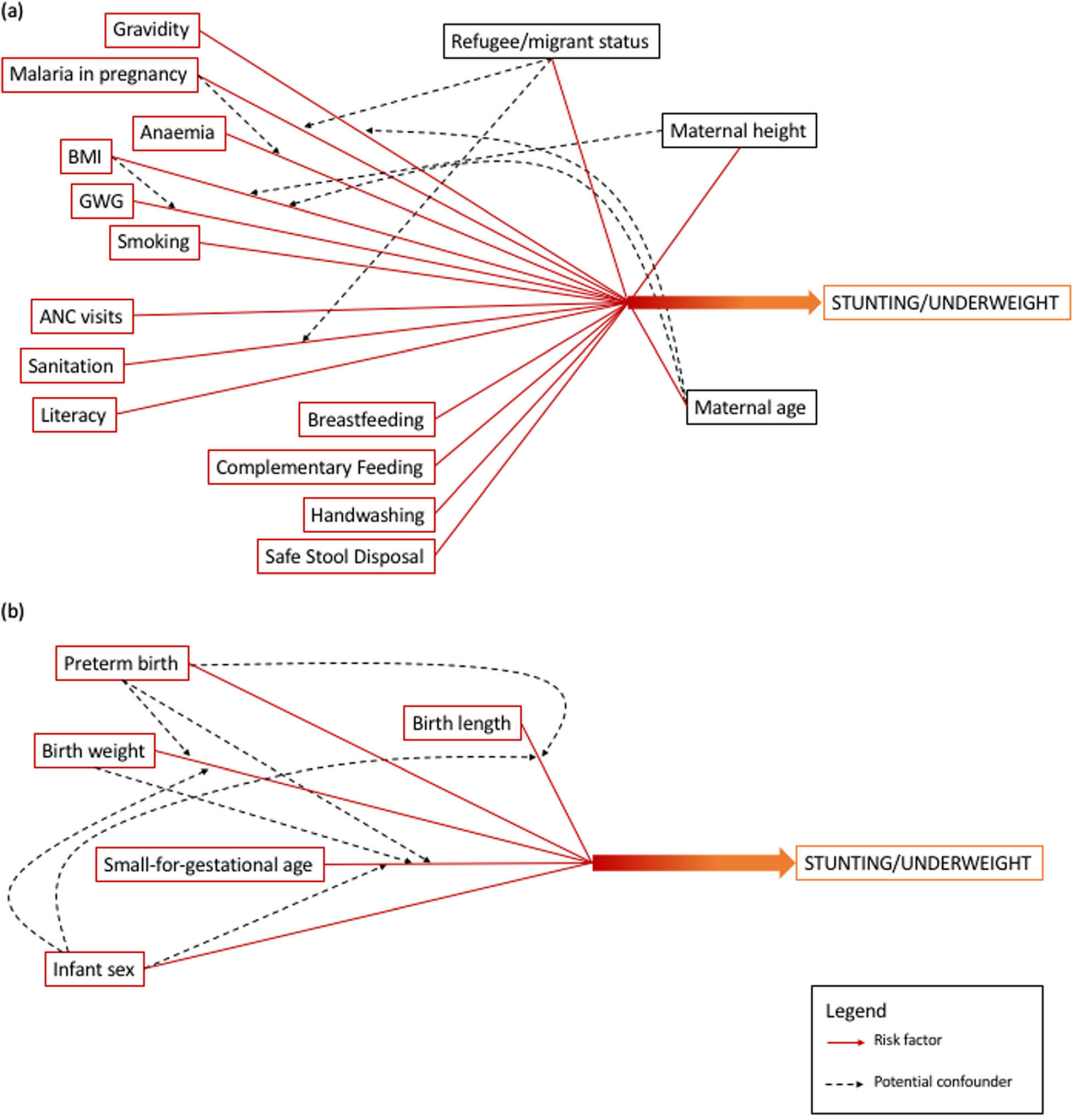
Conceptual model of risk factors and confounders and their relation to underweight and stunting by univariable and multivariable analysis. **a** Set of variables related to maternal risk factors; **b** set of variables related to infant risk factors

Univariable analysis of maternal variables and their relation to stunting and underweight included: maternal age, ethnicity (Karen or Burman); religion (Buddhist, Christian or Muslim); status (refugee or migrant); gravidity; maternal height; BMI category; gestational weight gain; malaria infection during pregnancy; anaemia; literacy; smoking status; number of ANC visits; length of stay at current residence; weekly food expenditure per household member; access to food resources (≥ 2 vs < 2 resources); sanitation; and time travelled to clinic. Maternal feeding practices included: breastfeeding (optimal vs suboptimal); minimum dietary diversity; minimum acceptable diet; any protein fed; handwashing; and safe stool disposal. Infant variables included: type of delivery; infant sex; birth weight; birth length; and infant age at time of cross-sectional survey. Four logistic regression models were then built to assess maternal variables and feeding practices (“maternal risk factor model”) and infant variables (“infant risk factor model”) and their effects on stunting and underweight. These models controlled for factors known to be related to stunting and underweight, as well as study site-specific variables and maternal feeding practices with a significance of *p* < 0.10 in univariable analysis. Figure 2 provides a simple schematic used to conceptualise the univariable and multivariable analysis performed. When used in these models, variables were transformed to provide more appropriate adjusted odds ratios, including: maternal height (unit = 5 cm); maternal age (unit = 1 year); and birth weight (unit = 50 g).

FGD were translated and transcribed with line-by-line open coding performed by two investigators (A.H. and K.P.) using NVivo version 10 and NVivo for Mac (QSR International, Doncaster, Victoria, Australia). *A priori* codes followed the IBM framework for elicitation, themes were characterized as attitudes (experiential and instrumental); normative influence and the motivation to comply; and personal agency made up of perceived control and self-efficacy. Where possible, these themes were further characterized by emergent themes related to infant malnutrition; infant feeding practices (exclusive breastfeeding, complementary feeding, dietary diversity, and meal frequency and amounts); and maternal nutrition and specifically, maternal under- and over-nutrition. AHH compiled these preliminary analyses, and with KP, codebooks were created and agreed upon, and FGD transcripts were re-analysed accordingly. AHH then performed thematic analysis [51] and reviewed findings with KP and the study team.

## Results

### Cross-sectional survey

Participation in the cross-sectional survey was high with 390/426 (91.5%) of all possible mothers interviewed. Demographic and household characteristics are presented in Table 2. Maternal age ranged between 17 and 46 years for all participants. One-third (132/390, 33.8%) of the infants were younger than 6 months. Across all sites, there was no significant difference in maternal or infant age; maternal or paternal education; or infant sex. Significant differences across sites included: ethnicity, religion, length of residence, weekly food expenditure per household member; access to ≥ 2 food resources; access to improved forms of sanitation; and time spent travelling to clinics (all variables significant for trends, *p* < 0.001). Although weekly food expenditure per household was lowest in refugees compared to migrants, refugees had the greatest proportion of women able to access ≥ 2 food resources, improved forms of sanitation and spent the least amount of time travelling to clinics.

**Table 2:** Demographic and household characteristics of 390 mothers enrolled in the cross-sectional component of the study by site

Maternal characteristics	Total	Refugees	Migrants	
		MLA	MKT	WPA
Total mothers surveyed, n (%)	390	165 (42.3)	112 (28.7)	113 (29.0)
Maternal age (yrs), mean (SD)	27.0 (6.4)	27.3 (6.3)	27.0 (6.8)	26.6 (6.2)
Paternal education (yrs), mean (SD)^a^	4.5 (3.7)	4.7 (4.0)	4.4 (3.6)	4.2 (3.4)
Infant age (months), mean (SD)	7.3 (3.4)	7.1 (3.6)	7.2 (3.3)	7.5 (3.4)
< 6 months, n (%)	132 (33.8)	62 (37.6)	37 (33.0)	33 (29.2)
≥ 6 months, n (%)	258 (66.2)	103 (62.4)	75 (67.0)	80 (70.8)
Infant sex, n (%)				
Male	179 (45.9)	79 (47.9)	52 (46.4)	48 (42.5)
Female	211 (54.1)	86 (52.1)	60 (53.6)	65 (57.5)
Ethnicity, n (%)^b^				
Karen	254 (65.1)	143 (86.7)	59 (52.7)	52 (46.0)
Burman	114 (29.2)	20 (12.1)	42 (37.5)	52 (46.0)
Other	22 (5.7)	2 (1.2)	11 (9.8)	9 (8.0)
Religion, n (%)^b^				
Buddhist	290 (74.4)	85 (51.5)	95 (84.8)	110 (97.3)
Christian	78 (20.0)	59 (35.8)	16 (14.3)	3 (2.7)
Muslim	21 (5.4)	21 (12.7)	0 (0)	0 (0)
Other	1 (0.2)	0 (0)	1 (0.9)	0 (0)
Literacy, n (%)				
Can read/write	263 (67.4)	116 (70.3)	77 (68.8)	70 (62.0)
Cannot read/write	127 (32.6)	49 (29.7)	35 (31.2)	43 (38.0)
Smoker, n (%)				
Yes	35 (9.0)	15 (9.1)	13 (11.6)	7 (6.2)
No	355 (91.0)	150 (90.9)	99 (88.4)	106 (93.8)
ANC visits, n (%)^c^				
< 8	23 (5.9)	11 (6.7)	6 (5.4)	6 (5.3)
≥ 8	366 (94.1)	154 (93.3)	105 (94.6)	107 (94.7)
Household characteristics				
Length of residence (yrs), mean (SD)^b^	7.6 (5.9)	9.3 (5.8)	7.0 (6.1)	5.8 (5.3)
Weekly food expenditure per household member (USD), mean (SD)^d^	4.3 (2.8)	3.6 (2.4)	4.8 (3.0)	4.7 (2.8)
≥ 2 food resources, n (%)^d^				
Yes	341 (87.9)	158 (96.3)	99 (89.2)	84 (74.3)
No	47 (12.1)	6 (3.7)	12 (10.8)	29 (25.7)
Sanitation, n (%)^e^				
Improved	362 (93.1)	163 (98.8)	94 (84.7)	105 (92.9)
Unimproved	27 (6.9)	2 (1.2)	17 (15.3)	8 (7.1)
Time travelled to clinic, n (%)^b^				
< 30 min	288 (73.9)	149 (90.3)	60 (53.6)	79 (69.9)
≥ 30 min	102 (26.1)	16 (9.7)	52 (46.4)	34 (30.1)

*MLA* Mae La refugee camp (“refugees”), *MKT* Mawker Thai village (“migrants”), *WPA* Wang Pha village (“migrants”), *ANC* antenatal care, *yrs* years, *SD* standard deviation

^a^“Unknown” data excluded: MKT (*n* = 101); WPA (*n* = 111)

^b^Significant for trend (*p* < 0.001)

^c^Missing data: MKT (*n* = 111)

^d^Significant for trend (*p* < 0.001). Missing data: MLA (*n* = 164); MKT (*n* = 111)

^e^Significant for trend (*p* < 0.001). Missing data: MKT (*n* = 111)

Table 3 summarises mothers’ practices for infant feeding, sanitation and hygiene. Three infants < 6 months (one at each site) were formula fed due to maternal preference and were excluded from this part of the analysis. Significant trends were noted for predominant and partial breastfeeding; feeding of safe water; minimum dietary diversity; minimum acceptable diet; handwashing; safe disposal of infant stool; and appropriate age of introduction of soft foods. For WASH practices, refugee mothers were notably better in all parameters compared to migrants. Although breastfeeding and appropriate introduction of soft foods were more adequate in refugees, minimum acceptable diet was notably lower compared to the migrant sites.

**Table 3:** Infant feeding practices of 390 mother-infant dyads enrolled in the cross-sectional component of study by site

Infant age < 6 months (*n* = 132), n (%)			
Breastfeeding^a^	Exclusive	Predominant^b^	Partial^c^
MLA (*n* = 61)	23 (37.7)	32 (52.5)	6 (9.8)
WPA (*n* = 32)	9 (28.1)	9 (28.1)	14 (43.8)
MKT (*n* = 36)	9 (25.0)	17 (47.2)	10 (27.8)
Infant age ≥ 6 months (*n* = 258), n (%)			
Practices	Safe water^c^	Dietary diversity^d^	Minimum acceptable diet^e^
MLA	94/98 (95.9)	23/103 (22.3)	2/103 (1.9)
WPA	62/79 (78.5)	12/80 (15.0)	7/80 (8.8)
MKT	47/73 (64.4)	23/75 (30.7)	9/75 (12.0)
All infants (*n* = 390), n (%)			
Practices	Handwashing^c^	Safe stool disposal^c^	Appropriate age food introduction^c^
MLA	151/157 (96.2)	84/161 (52.2)	141/165 (85.5)
WPA	79/107 (73.8)	51/105 (48.6)	60/113 (53.1)
MKT	62/102 (60.8)	18/89 (20.2)	55/112 (49.1)

*MLA* Mae La refugee camp, *MKT* Mawker Thai village, *WPA* Wang Pha village

^a^Excludes three mothers (one from each site) who only fed formula milk

^b^Significant for trend (*p* = 0.029)

^c^Significant for trend (*p* < 0.001). “Safe water” excludes 5 mothers from MLA, 1 from WPA, and 1 from MKT who did not feed water the day prior. “Handwashing” excludes 4 mothers from MLA, 6 from WPA, and 10 from MKT who did not prepare the food the day prior

^d^Significant for trend (*p* = 0.020)

^e^Significant for trend (*p* = 0.008)

Table 4 presents results of univariable and multivariable logistic regression analysis for maternal and infant risk factors and their association with stunting and underweight (for full analysis, refer to Additional file 2: Table S1). Overall, 17.9% (70/390) of infants were stunted and 14.9% (58/390) were underweight. Although decreased gestational weight gain and handwashing were significantly associated with both stunting and underweight, these variables were not included in multivariable regression analysis as they greatly limited the sample size. Collinear relationships included: refugee or migrant status and malaria in pregnancy, form of sanitation, time travelled to the clinic and safe disposal of infant stool; and small-for-gestational-age with infant birth weight and birth length. Each 5 cm increase in maternal height had decreased odds of stunting (AOR: 0.50, 95% CI: 0.38, 0.66, *p* < 0.001) and underweight (AOR: 0.64, 95% CI: 0.48, 0.85, *p* = 0.002), while stunting (AOR: 2.08, 95% CI: 1.12, 3.84, *p* = 0.020) and underweight (AOR: 2.26, 95% CI: 1.17, 4.36, *p* = 0.015) had increased odds among migrants. When adjusting for length of gestation, infant sex and infant age, only small-for-gestational age was associated with an increased odds of stunting (AOR: 3.42, 95% CI: 1.88, 6.22, *p* < 0.001) and underweight (AOR: 4.44, 95% CI: 2.36, 8.34, both *p* < 0.001).

**Table 4:** Maternal and infant variables of interest from cross-sectional survey and regression analysis

Variables of interest					Univariable (*p*-value)		Adjusted OR, (95% CI); *p*-value	
	Stunting (*n* = 70)	Normal (*n* = 320)	Underweight (*n* = 58)	Normal (*n* = 332)	Stunting	Underweight	Stunting	Underweight
Maternal^a^								
Age (yrs), mean (SD)	27.3 (6.8)	27.0 (6.3)	28.6 (7.3)	26.7 (6.2)	0.707	0.036	1.01, (0.97, 1.06); 0.602	1.06, (1.01, 1.10); 0.019
Status, n (%)								
Refugee (Referent)	21 (12.7)	144 (87.3)	16 (9.7)	149 (90.3)	0.021	0.014	2.08, (1.12, 3.84); 0.020	2.26, (1.17, 4.36); 0.015
Migrant	49 (21.8)	176 (78.2)	42 (18.7)	183 (81.3)				
Height (cm), mean (SD)^b^	148.0 (5.2)	151.6 (5.2)	149.1 (5.7)	151.3 (5.2)	< 0.001	0.003	0.50, (0.38, 0.66); < 0.001	0.64, (0.48, 0.85); 0.002
Malaria in pregnancy, n (%)								
Yes	12 (34.3)	23 (65.7)	10 (28.6)	25 (71.4)	0.008	0.017	1.88, (0.79, 4.45); 0.151	1.91, (0.80, 4.57); 0.145
No (Referent)	58 (16.3)	297 (83.7)	48 (13.5)	307 (86.5)				
Literacy, n (%)								
Can read/write	41 (15.6)	222 (84.4)	33 (12.6)	230 (87.5)	0.081	0.063	0.98, (0.52, 1.85); 0.961	0.87, (0.47, 1.62); 0.668
Cannot read/write (Referent)	29 (22.8)	98 (77.2)	25 (19.7)	102 (80.3)				
Smoker, n (%)								
Yes	11 (31.4)	24 (68.6)	7 (20.0)	28 (80.0)	0.029	0.371	1.80, (0.70, 4.62); 0.224	Not included
No (Referent)	59 (16.6)	296 (83.4)	51 (14.4)	304 (85.6)				
Infant^c^								
Length of gestation, n (%)								
Term (Referent)	60 (16.6)	302 (83.4)	53 (14.6)	309 (85.4)	0.011	0.587	3.05, (1.29, 7.22); 0.011	1.35, (0.47, 3.91) 0.580
< 37 wks	10 (35.7)	18 (64.3)	5 (17.9)	23 (82.1)				
Sex, n (%)								
Male	41 (22.9)	138 (77.1)	34 (19.0)	145 (81.0)	0.019	0.035	1.87, (1.08, 3.23); 0.025	1.94, (1.07, 3.52); 0.029
Female (Referent)	29 (13.7)	182 (86.3)	24 (11.4)	187 (88.6)				
Small-for-gestational age, n (%)								
Yes	26 (32.1)	55 (67.9)	25 (30.9)	56 (69.1)	< 0.001	< 0.001	3.42, (1.88, 6.22); < 0.001	4.44, (2.36, 8.34); < 0.001
No (Referent)	44 (14.7)	256 (85.3)	33 (11.0)	267 (89.0)				
Age (months), mean (SD)	7.8 (3.5)	7.2 (3.4)	8.2 (3.6)	7.1 (3.4)	0.194	0.025	1.06, (0.98, 1.15); 0.121	1.12, (1.03, 1.22); 0.008

For all variables included in univariable analysis, please refer to Additional file 2: Table S1

A total of 4 multivariable regression models were created: 1 model tested “maternal risk factors” and infant feeding and WASH behavioral factors and 1 model tested “infant risk factors”, with both models tested for association with stunting and underweight

^a^Total number of observations included in multivariable regression for “maternal risk factors” for stunting (*n* = 389) and underweight (*n* = 381). Adjusted for maternal age

^b^Unit of 5 cm used for multivariable regression

^c^Total number of observations included in multivariable regression for “infant risk factors” for stunting and underweight (*n* = 381). Adjusted for length of gestation, infant sex and infant age

^d^A total of 9 data points missing (*n* = 381)

### Focus group discussions

Of the 10 FGD conducted, 3 were performed in MLA, 3 in WPA, and 4 in MKT. A total of 63 women participated. Ages ranged from 18 to 35 years for all FGD. FGD included Sgaw Karen, Pwo Karen and Burman. Please refer to Additional file 3: Table S2 for full quotes as they relate to key themes.

#### Attitudes (experiential and instrumental)

##### Infant malnutrition

Many women demonstrated limited knowledge about chronic malnutrition, which may have been due to a genuine lack of awareness or simply a lack of this term in either Karen or Burmese. “Nutrition” was often described in terms of “energy,” likely related to a direct translation of healthy foods being “foods full of energy.” This was commonly mentioned and reflected in conversations assessing attitudes and beliefs toward infant malnutrition. Women discussed malnutrition as a constellation of symptoms suggestive of acute malnutrition in children.

Participant 1: “My neighbor’s child became sick and then became malnourished, losing a lot of weight. Her body became very thin so that the joints in her arms and legs became prominent (visible).”

Participant 2: “I saw one child who had a big belly and you could see every rib; the arms and legs were thin and the baby could not walk—he could only lay on the bed.”—Karen multiparous women from MLA

“Sometimes the parents don’t feed their baby enough food [ … ]. If the baby receives enough food then they get energy and if it is not enough then the baby can’t grow up well. [ … ] Malnutrition is a kind of energy. Children can’t grow up because the food that they eat does not turn into energy.”—Karen multiparous woman from MKT

##### Infant feeding practices

Attitudes and beliefs related to breastfeeding, complementary feeding, dietary diversity, and meal frequency and amounts demonstrated strong explanatory power for rates of appropriate infant feeding observed in the cross-sectional component of this study. Predominant breastfeeding, with water in addition to breast milk, was reported in all FGD. The rationale for this practice included: preventing thirst in the infant, difficulties in suckling and “dry throat.” Women would often cite “heartburn,” resembling infantile colic, as a reason for feeding water. Women also claimed that their infants could be soothed by feeding soft foods prior to 6 months, implying partial breastfeeding could begin as early as 2 months of age—as seen in the cross-sectional component as well.

“To ‘cool’ the baby’s stomach because the baby has colic [mother’s perception that the baby is hungry]. We do to help the baby get healthy and strong. I don’t give much food, just one or two spoons. From 3 months until 6 months, I give only rice. After 6 months I give other foods.”—Burman multiparous woman from MKT

Discussions also suggested attitudes and beliefs that could explain the low rates of dietary diversity and minimum acceptable diet observed in the cross-sectional survey. Rice and fruits, such as bananas, were deemed appropriate for complementary feeding. Feeding “snacks” such as processed foods readily available in the local markets (USD $0.15) was widespread. These “soft foods” were fed to infants given their ease of mechanical digestion, low cost, accessibility and “mollifying” effects. There was great variability in when mothers introduced soft foods to their infants’ diets. Most mothers felt that from 6 to 9 months, foods soft in texture should be provided to infants given ease of mechanical digestion. Across all sites and ethnicities, mothers reported waiting until 9 to 12 months before feeding proteinaceous foods, as infants lacked teeth and risked worm infections if fed meat “too early.” Some mothers introduced soft foods when the infant reached certain developmental milestones, e.g., “when they can take food for themselves” or “when the child can sit.” Although rare before 9 months, the predominant source of protein was eggs and, less frequently, beans or lentils. As one participant explained:

“I think it is ok to feed potato but not meat and fish because the baby is too young and I’m afraid the baby might get worms. If the baby has worms then he/she might have stomach pain or discomfort.”—Burman nulliparous woman from MKT

In addition to dietary diversity, meal frequencies and amounts were also quite variable—potentially explaining the low rates of minimum acceptable diet observed in the cross-sectional survey.

Participant 1: “We give the food twice a day, three soup spoonfuls. We give this way until one year. 6 months to one year we give three spoons, and 6 months to 8 months we also give three spoons.”

Participant 2: “I think that it is not enough food. For me, I would increase every month. I did not learn from anywhere; I think that if the baby is getting older that their stomach is becoming bigger. I give a different kind of a spoon, but I start feeding with 3 soup spoons then increase monthly and start feeding twice a day.”—Burman nulliparous women from WPA

##### Maternal nutrition

Women were able to express their attitudes about maternal nutrition: particularly outcomes related to under- and over-nutrition, their effects on the growing fetus, and healthy eating during pregnancy.

Participant 1: “The pregnant women who become malnourished may affect the baby who will not have energy. It may be easy for the baby to become ill.”

Participant 2: “If you don’t eat, you may have a premature delivery or your baby will have low birth weight.”—Burman multiparous women from MKT

“If we eat too much and become too fat then our uterus might contract and it becomes more difficult to give birth. If you are too fat, you can’t give birth when the uterus is contracted, you have to deliver through cesarean section delivery.”—Karen multiparous woman from MKT

#### Normative influence and motivation to comply

##### Infant feeding practices

Normative influences were readily discussed by participants concerning infant feeding practices. Participants acknowledged that the primary sources of information on feeding practices included mothers, aunts, siblings and older women in the community. Mothers with appropriate practices often quoted flyers or posters found at health facilities or relied on family members working in health care settings. Comments also suggested a woman’s motivation to comply with the information she received, often based on the perceived trustworthiness of the source.

“The meat may be raw and smell bad. I heard from older women that you can’t give the woman who has just delivered fresh meat or fish because it may be harmful. [ … ] I don’t give meat to my child very often, but my younger sister gave everything and the child had many episodes of diarrhea. She made different foods for her child like fish, chicken but not beef and pork because it’s too hard for the child. But the baby could take only a little bit.”—Karen multiparous woman from MLA

#### Personal agency: perceived control and self-efficacy

##### Infant malnutrition

Under-nutrition—among infants as well as mothers—was readily associated with poverty. Self-efficacy in ensuring appropriate infant feeding was limited by a lack of awareness among parents.

“I have two malnourished babies; both were born early at 7 months. I fed breast milk but the baby did not look strong. The baby has been very thin since was born until now. I fed breast milk but still the baby did not gain weight. I think this is because of many kinds of problems I face [poverty]. That’s why I buy medicine or vitamin for the baby but still, he is malnourished. Both the mother and the baby don’t have good nutrition; my baby is now 7 years old and still very thin and not growing.”—Muslim woman from MLA

##### Infant feeding practices

Perceived barriers (“control”) and overcoming these barriers (“self-efficacy”) could also influence breastfeeding and complementary feeding practices. For example, early introduction of water and soft foods related to overcoming a commonly perceived barrier: insufficient breast milk production. Dietary diversity, and particularly meat as a source of protein, was often not fed due to a perceived lack of control over an infant’s food preferences.

“Some of the babies don’t like meat; if they don’t like meat, have to make with eggs or potatoes.”

“Like you said, my baby does not like meat, I give but they don’t like. Now until 4 years they don’t like meat. [ … ] Fish they don’t eat.”—Muslim women from MLA

In addition, dietary diversity and minimum acceptable diet were limited by a lack of awareness or unclear use or understanding of “developmental milestones” to determine appropriate timing of food introduction, with the age of 1 year often cited as a crucial point in an infants’ life that signalled changes in diet. An illustrative example:

“I never feed my baby the same way as the other mothers feed. Some mothers even feed their baby with hard rice when the baby is only 6-7 months. For me when my baby is 6-7 months, I have not fed my baby with rice yet. I feed only after my baby is one year old. [ … ] I cannot remember exactly which month I can start these types of foods, it is not like that. It depends on the period when the baby can eat or cannot eat yet. I feed anything that the baby can eat.”—Burman multiparous woman from WPA

Finally, the need to work limited a woman’s perceived control over appropriately feeding her infant.

“If we travel we have to bring food along with us so I wait until he can eat by himself. But snacks are easy for him to take and he can have it at any time. So I start to give the food around 4 or 5 months.”—Karen multiparous woman from MLA

When probed however, women suggested that any barriers in preparing soft foods for the infant were easily overcome, given the prevailing belief that feeding an infant appropriately was paramount to any perceived difficulties.

Participant 1: “It is not difficult because this is our habit, every day.”

Participant 2: “This is only for our babies so it is not difficult because we have to feed our babies.”

Participant 3: “If it is for our baby, we can do it, it is not difficult.”—Burman multiparous women from MKT

##### Maternal nutrition

Maternal under-nutrition, as alluded to above, was linked with poverty among many participants.

Participant 1: “The women who don’t have much money, if they spend on food their money will be gone. If they eat a little bit one day, they may keep food for the next day.

Participant 2: “For me, when I was pregnant, food was expensive, if I get a little bit, I am satisfied.”—Burman multiparous women from MKT

Additional barriers impacting participants’ perceived control over their own diet during pregnancy related to age, comorbidities from chronic disease, and appetite as this quote suggests:

“During my last pregnancy, I had anemia so the baby also got anemia. [ … ] Now, as pregnant women who are older—more than 30 or 40 years—we cannot eat well like when we were young. If we do not eat well, we cannot make good breast milk. Now, if I deliver this baby I will not have enough breast milk because of my age so my baby will also not be that strong. [ … ] Maybe I have enough breast milk, but anemia is also bad for my breast milk and for my baby. [ … ] Because I am not so poor, I can buy food, but even if I eat, I have low sugar and still have poor nutrition.”—Muslim woman from MLA

Finally, it bears mentioning that participant knowledge of a healthy diet in pregnancy was limited as demonstrated in another study exploring perceptions of maternal nutrition in these populations, with implications for self-efficacy in ensuring good nutrition during pregnancy [52].

## Discussion

To the best of our knowledge, this study is the first in-depth look at the infant feeding practices among mothers in refugee and migrant communities along the Thailand-Myanmar border. This study is in line with existing literature that increasingly links maternal health and nutrition to infant nutrition [53–55], where factors such as maternal age, maternal height, malaria in pregnancy, smoking, gestational weight gain and being born small-for-gestational-age, remain important concerns in these marginalised communities and integral to the overall health of infants.

The two components of the mixed-methods approach identified inadequacies in infant feeding practices. The cross-sectional survey showed high rates of suboptimal breastfeeding and inadequate infant feeding similar to those in neighbouring Myanmar [14, 15]. The proportion of stunting in infants less than 1 year of age matches annual surveys of children across all refugee camps along the Thailand-Myanmar border [12] and confirms high rates of chronic malnutrition in the first year of life. Maternal infant feeding practices showed only marginal effects on stunting and underweight when controlling for potential confounders. Improved infant nutritional outcomes in refugee compared to migrant sites may reflect better access to health education and social support services provided by non-governmental organisations [56] and greater proximity to markets in the refugee camps. Furthermore, the mixed-methods design allowed qualitative findings to help explain the results of the cross-sectional survey. For example, although refugees seem to have more appropriate introduction of soft foods for infant diet, refugee mothers provide their infants with diets of limited diversity and adequacy. In addition, migrants were more likely to report partial breastfeeding, which suggests that they have increased dietary diversity and minimum acceptable diet not because of awareness, but rather as it relates to a mother’s experience of providing an infant soft foods too soon. The ability and need for migrant women to work compared to refugees who are legally precluded from work outside the refugee camps in Thailand may also impact rates of exclusive breastfeeding and partial breastfeeding observed in cross-section.

FGD drew connections to social barriers similar to those described in other studies: poverty (e.g., limited income generation and irregular employment); food insecurity and limited food resources; and geographical, language, and ethnic or minority status [57–62]. Such differences may also relate to the significantly lengthier duration of residence in the refugee camp compared to the migrant communities. Hence, migrant status likely encompasses a range of social determinants impacting poor nutritional outcomes in these marginalised communities, but we were unable to further characterise this due to the limited sample size. In providing evidence for contextual factors and their importance, a recent study on stunting in Thailand using survey data from 2012 pointed out the important interaction between prolonged duration of breastfeeding (breastfeeding after 12 months) and impoverished households, positing a link between poverty and an inability to provide appropriate complementary feeding after 6 months of age [25]. In the current study, “migrant status” may be considered a proxy for limited access to food resources and improved forms of sanitation, lower rates of safe disposal of infant stool and inappropriate handwashing—important indicators of relative poverty with potential impacts on infant nutrition and health. Therefore, we view this study as complementary to the aforementioned study from Thailand, as findings presented here describe important, plausible mechanisms by which poverty and inadequate complementary feeding interact and lead to the rates of infant chronic malnutrition observed [25].

Although demographics and infant feeding or sanitation practices differ across sites, results of our regression analysis suggest that these differences are small in comparison to contextual and proximal factors related to stunting and underweight. However, the research literature emphasises the importance of feeding practices in infancy, particularly as the growing infant affords greater distance from the risks attributable to maternal nutrition, pregnancy and birth. Therefore, the design of this study proved important—not only do we report rates of infant feeding behaviours and their relation to risk factors for malnutrition, but findings also assess the local attitudes, beliefs and an individuals’ ability to adequately feed their infants. As improvements in maternal health occur in line with population-level improvements in infant feeding and sanitation practices, the qualitative component of this study adds important understanding of local rationale behind maternal behaviours that can impact infant nutrition. Mothers’ reasons for these behaviours were consistent with those documented among mothers throughout South and Southeast Asia [57, 59, 62–68]. As many studies with similar findings extend back to the turn of the century, it is impressive to note that mothers’ understanding and rationale have remained unchanged over time. Importantly, mothers frequently learn how to feed infants from other women in the community: mothers, aunts, elderly women and neighbours with children. Findings from this study demonstrate limited understanding and awareness of healthy diets during pregnancy that may lead to improved outcomes in newborns, corroborating findings in a recent study focused predominantly on maternal nutrition [52]. However, as one of the first studies utilising the IBM framework, we add to the literature by providing a firmer grasp of how these findings may lend to culturally appropriate counselling measures.

Although strengthened by a mixed-methods approach, this study has limitations. In spite of high participation in the cross-sectional survey, analysis is limited as it is not prospective in nature and assumes that infant feeding practices have persisted since birth. The cross-sectional design may not sufficiently quantify inappropriate feeding practices and does not allow for following these exposures until infants reach 2 years of age. A recent cohort study demonstrated highly variable rates of breastfeeding “exclusivity” that the present study was unable to capture [69], which limits our characterisation of complementary feeding practices in this study population. Therefore, future, controlled, large prospective studies would yield clearer associations between maternal and infant risk factors for infant undernutrition. It would behove future studies to look more systematically at attitudes and beliefs about infant feeding practices in this population, as the purpose of the qualitative portion here was to explain findings from the cross-sectional survey, and not specifically designed to quantify the extent to which women in these communities hold these beliefs.

### Implications

A number of implications—at the community level as well as for research and health programming—emerge from this study’s findings. Future research to improve nutritional status of refugee and migrant infants should pay heed to social constraints and evaluate the effects of limited economic opportunity and access to food resources [61]. Infant nutrition programs should consider home-based visits and community mobilisation activities that involve older, experienced mothers providing reliable and consistent information about child feeding [70, 71]. Indeed, based on findings from this study, a small, home-based counselling intervention was piloted in the same refugee population studied here, demonstrating improved rates of exclusive breastfeeding, minimum dietary diversity, minimum acceptable diet and appropriate sanitation practices [72]. However, further research is needed to identify the cost-effectiveness of home-based counselling for infant feeding and WASH practices in these communities, especially given the highly mobile nature of the migrant population along the border. As the greatest improvement in child health points to care during pregnancy, research should also seek to characterise maternal nutritional status or test appropriate interventions and determine their relationship to long-term child nutrition. Interventions targeting reproductive-aged women for improved infant nutritional outcomes may include: counselling for exclusive breastfeeding during the antenatal period; family planning interventions made available for older women; post-partum weight reduction counselling; and micronutrient and balanced protein supplementation during and prior to pregnancy [52, 54, 73–77]. Population- and community-level public health campaigns may generate awareness of appropriate infant feeding practices and improve access to healthy foods and sanitation, with such efforts enhanced through appropriate policies (organisational, institutional or governmental) that target social determinants that affect these disadvantaged groups.

## Conclusions

Inadequate infant feeding practices are widespread in refugee and migrant communities along the Thailand-Myanmar border. Risk factors particular to maternal nutrition and infant birth should be considered for future programming to reduce the burden of chronic malnutrition in infants.

## Supplementary information

**Additional file 1.** Focus group discussion guide.

**Additional file 2: Table S1.** Full regression analysis.

**Additional file 3: Table S2.** Additional quotes from focus group discussions.

## Data Availability

Data and materials are available upon request to the corresponding author, given sensitivities to the migrant and refugee status of the women participating in this study.

## Abbreviations

ANC: Antenatal care
BMI: Body mass index
FGD: Focus group discussion(s)
LMIC: Low- and middle-income countries
MKT: Mawker Thai
MLA: Mae La refugee camp
SD: Standard deviation
SMRU: Shoklo Malaria Research Unit
WPA: Wang Pha
