# Supplementary material for: Feeding practices and risk factors for chronic infant undernutrition among refugees and migrants along the Thailand-Myanmar border: a mixed-methods study
Source: BMC Public Health. 2019 Nov 28;19:1586. doi: 10.1186/s12889-019-7825-7 (PMC6883662; doi:10.1186/s12889-019-7825-7)
Supplement: Supplementary file 1 — Additional file 1. Focus group discussion guide. [file 12889_2019_7825_MOESM1_ESM.pdf]

Additional file 3: Focus group discussion guide for "Feeding practices and risk factors for chronic infant undernutrition among refugees and migrants along the Thailand-Myanmar border: a mixed-methods study".

1. What is malnutrition?
    a. Who is affected?
    b. *Probe: Can pregnant women have this problem? What can happen?*
2. Have you heard of overnutrition or overweight? What is it?
    a. Who is affected? How?
    b. *Probe: Can this be a problem for children? What can happen?*
3. During pregnancy, what are the good foods to eat?
    a. How much should you eat every day? Does this change as you get closer to delivery? After delivery? How? Why?

Now, I'd like to ask you some questions about how to feed your baby.

4. What is the best way to feed your baby after delivery?
    a. Why?
    b. *Probe: Is it OK to feed water? When? Why?*
5. When should you start feeding the baby soft foods or rice?
    a. *Probe: if you get answers before 6 months: why? Where did you learn this?*
6. What are the good foods to feed baby?
    a. Why? *Probe: does anyone have other ideas? Where did you learn this?*
    b. *Probe if you get answers like fruit: why?*
    c. *Probe: what about feeding other foods like meat, egg, fruits, vegetables? When should baby start eating these foods?*
7. How do you prepare soft foods or rice for your baby?
    a. What are the difficulties in feeding the baby when you start feed foods?
        i. *Probe: preparing the food—making it soft and easy for baby to eat?*
        ii. *Probe: time it takes to prepare food?*
        iii. *Probe: baby doesn't want to eat? Baby takes too long to feed?*
    b. How do you prepare foods for the baby?
        i. *Probe: what are the best ways to make the food soft so baby can eat easy?*
8. How much should you feed baby? How many meals should you give baby every day?
    a. *Probe: when you start feeding soft food or rice*?
    b. *Probe*: *Does this change when the baby gets older, like at 6 months or 12 months? Why? How?*

End the session with counseling on feeding during pregnancy and for the child.
