# Supplementary material for: Feeding practices and risk factors for chronic infant undernutrition among refugees and migrants along the Thailand-Myanmar border: a mixed-methods study
Source: BMC Public Health. 2019 Nov 28;19:1586. doi: 10.1186/s12889-019-7825-7 (PMC6883662; doi:10.1186/s12889-019-7825-7)
Supplement: Supplementary file 2 — Additional file 2: Table S1. Full regression analysis. [file 12889_2019_7825_MOESM2_ESM.pdf]

Table S1. Maternal and infant variables of interest from cross-sectional survey and regression analysis.

Variables of interest	Stunting (n=70)	Normal (n=320)	Underweight (n=58)	Normal (n=332)	Univariable (p-value)		Adjusted OR, (95% CI); p-value	
					Stunting	Underweight	Stunting	Underweight
**Maternal**[a]								
Age (yrs), mean (SD)	27.3 (6.8)	27.0 (6.3)	28.6 (7.3)	26.7 (6.2)	0.707	0.036	1.01, (0.97, 1.06); 0.602	1.06, (1.01, 1.10); 0.019
Ethnicity, n (%)								
Karen	47 (18.5)	207 (72.1)	35 (13.8)	219 (86.2)	0.401	0.612	Not included	Not included
Burman	17 (14.9)	97 (85.1)	18 (15.8)	96 (84.2)				
Religion, n (%)								
Buddhist	55 (19.0)	235 (81.0)	48 (16.6)	242 (83.5)	0.283	0.102	Not included	Not included
Christian	11 (14.1)	67 (85.9)	9 (11.5)	69 (88.5)				
Muslim	3 (14.3)	18 (85.7)	1 (4.76)	20 (95.2)				
Status, n (%)								
Refugee (Referent)	21 (12.7)	144 (87.3)	16 (9.7)	149 (90.3)	0.021	0.014	2.08, (1.12, 3.84); 0.020	2.26, (1.17, 4.36); 0.015
Migrant	49 (21.8)	176 (78.2)	42 (18.7)	183 (81.3)				
Gravidity, n (%)								
Primigravida	18 (14.2)	109 (85.8)	15 (11.8)	112 (88.2)	0.177	0.238	Not included	Not included
Multigravida	52 (19.8)	211 (80.2)	43 (16.4)	220 (83.7)				
Height (cm), mean (SD)[b]	148.0 (5.2)	151.6 (5.2)	149.1 (5.7)	151.3 (5.2)	<0.001	0.003	0.50, (0.38, 0.66); <0.001	0.64, (0.48, 0.85); 0.002
BMI, n (%)[c]								
Low (< 18.5 kg/m^2)	10 (17.0)	49 (83.1)	11 (18.6)	48 (81.4)	0.974	0.434	Not included	Not included
Normal (18.5-22.9 kg/m^2)	27 (15.7)	145 (84.3)	24 (14.0)	148 (86.0)				
High (≥ 23 kg/m^2)	12 (16.2)	62 (83.8)	8 (10.8)	66 (89.2)				
GWG, n (%)[d]								
< 10 kg	29 (21.0)	109 (79.0)	24 (17.4)	114 (82.6)	0.018	0.041	Not included	Not included
10-15 kg	19 (14.8)	109 (85.2)	18 (14.1)	110 (85.9)				
≥ 15 kg	2 (5.0)	38 (95.0)	1 (2.5)	39 (97.5)				
Malaria in pregnancy, n (%)								
Yes	12 (34.3)	23 (65.7)	10 (28.6)	25 (71.4)	0.008	0.017	1.88, (0.79, 4.45); 0.151	1.91, (0.80, 4.57); 0.145
No (Referent)	58 (16.3)	297 (83.7)	48 (13.5)	307 (86.5)				
Anemia, n (%)								
Yes	16 (19.5)	66 (80.5)	10 (12.2)	72 (87.8)	0.678	0.443	Not included	Not included
No	54 (17.5)	254 (82.5)	48 (15.6)	260 (84.4)				
Literacy, n (%)								
Can read/write	41 (15.6)	222 (84.4)	33 (12.6)	230 (87.5)	0.081	0.063	0.98, (0.52, 1.85); 0.961	0.87, (0.47, 1.62); 0.668
Cannot read/write (Referent)	29 (22.8)	98 (77.2)	25 (19.7)	102 (80.3)				
Smoker, n (%)								
Yes	11 (31.4)	24 (68.6)	7 (20.0)	28 (80.0)	0.029	0.371	1.80, (0.70, 4.62);	Not included

Variables of interest	Stunting (n=70)	Normal (n=320)	Underweight (n=58)	Normal (n=332)	Univariable (p-value)		Adjusted OR, (95% CI); p-value	
					Stunting	Underweight	Stunting	Underweight
No (Referent)	59 (16.6)	296 (83.4)	51 (14.4)	304 (85.6)			0.224	
ANC visits, n (%)								
<8 visits (Referent)	6 (26.1)	17 (73.9)	5 (21.7)	18 (78.3)	0.274	0.363	Not included	Not included
≥8 visits	64 (17.5)	302 (82.5)	53 (14.5)	313 (85.5)				
Length of residence (yrs), mean (SD)	8.3 (6.4)	7.5 (5.8)	7.8 (6.1)	7.6 (5.9)	0.278	0.781	Not included	Not included
Weekly food expenditure per household member (USD), mean (SD)	4.19 (2.62)	4.29 (2.78)	4.00 (2.25)	4.32 (2.83)	0.773	0.417	Not included	Not included
≥2 food resources, n (%)								
Yes	62 (18.2)	279 (81.8)	49 (14.4)	292 (85.6)	0.846	0.389	Not included	Not included
No	8 (17.0)	39 (83.0)	9 (19.2)	38 (80.9)				
Sanitation, n (%)[e]								
Improved (Referent)	62 (17.1)	300 (82.9)	51 (14.1)	311 (85.9)	0.103	0.096	Not included	Not included
Unimproved	8 (29.6)	19 (70.4)	7 (25.9)	20 (74.1)				
Time travelled to clinic, n (%)[e]								
<30 minutes (Referent)	43 (14.9)	245 (85.1)	33 (11.5)	255 (88.5)	0.009	0.001	Not included	Not included
≥30 minutes	27 (26.5)	75 (73.5)	25 (24.5)	77 (75.5)				
**Infant[f]**								
Length of gestation, n (%)								
Term (Referent)	60 (16.6)	302 (83.4)	53 (14.6)	309 (85.4)	0.011	0.587	3.05, (1.29, 7.22); 0.011	1.35, (0.47, 3.91) 0.580
<37 wks	10 (35.7)	18 (64.3)	5 (17.9)	23 (82.1)				
Sex, n (%)								
Male	41 (22.9)	138 (77.1)	34 (19.0)	145 (81.0)	0.019	0.035	1.87, (1.08, 3.23); 0.025	1.94, (1.07, 3.52); 0.029
Female (Referent)	29 (13.7)	182 (86.3)	24 (11.4)	187 (88.6)				
Birth weight (kg), mean (SD)[g]	2.620 (0.45)	2.990 (0.44)	2.650 (0.46)	2.970 (0.45)	<0.001	<0.001	Not included	Not included
Birth length (cm), mean (SD)[g]	46.2 (3.1)	48.5 (2.2)	46.9 (2.6)	48.3 (2.5)	<0.001	<0.001	Not included	Not included
Small-for-gestational age, n (%)[h]								
Yes	26 (32.1)	55 (67.9)	25 (30.9)	56 (69.1)	<0.001	<0.001	3.42, (1.88, 6.22); <0.001	4.44, (2.36, 8.34); <0.001
No (Referent)	44 (14.7)	256 (85.3)	33 (11.0)	267 (89.0)				
Age (months), mean (SD)	7.8 (3.5)	7.2 (3.4)	8.2 (3.6)	7.1 (3.4)	0.194	0.025	1.06, (0.98, 1.15); 0.121	1.12, (1.03, 1.22); 0.008
**Practices[a]**								
Breastfeeding, n (%)								
Optimal	5 (12.2)	36 (87.8)	4 (9.76)	37 (90.2)	0.452	0.580	Not included	Not included
Suboptimal	17 (19.3)	71 (80.7)	13 (14.8)	75 (85.2)				
Dietary diversity, n (%)								
≥ 4 food groups fed	13 (22.4)	45 (77.6)	13 (22.4)	45 (77.6)	0.334	0.189	Not included	Not included
< 4 food groups fed	39 (17.0)	191 (83.0)	35 (15.2)	195 (84.8)				
Minimum acceptable diet, n (%)								

Variables of interest	Stunting (n=70)	Normal (n=320)	Underweight (n=58)	Normal (n=332)	Univariable (p-value)		Adjusted OR, (95% CI); p-value	
					Stunting	Underweight	Stunting	Underweight
Yes	3 (16.7)	15 (83.3)	4 (22.2)	14 (77.8)	1.00	0.500	Not included	Not included
No	43 (17.9)	197 (82.1)	37 (15.4)	203 (84.6)				
Any protein fed, n (%)								
Yes	18 (19.8)	73 (80.2)	17 (18.7)	74 (81.3)	0.465	0.366	Not included	Not included
No	27 (16.2)	140 (83.8)	24 (14.4)	143 (85.6)				
Handwashing, n (%)[e]								
Yes	43 (14.7)	249 (85.3)	37 (12.7)	255 (87.3)	0.025	0.051	Not included	Not included
No (Referent)	19 (25.7)	55 (74.3)	16 (21.6)	58 (78.4)				
Safe stool disposal, n (%)[e]								
Yes (Referent)	25 (16.3)	128 (83.7)	15 (9.8)	138 (90.2)	0.806	0.044	Not included	Not included
No	35 (17.3)	167 (82.7)	35 (17.3)	167 (82.7)				

A total of 4 multivariable regression models were created: 1 model tested “maternal risk factors” and infant feeding and WASH behavioral factors and 1 model tested “infant risk factors”, with both models tested for association with stunting and underweight.

[a]Total number of observations included in multivariable regression for “maternal risk factors” for stunting (n=389) and underweight (n=381). Adjusted for maternal age.

[b]Unit of 5 cm used for multivariable regression.

[c]Included only those women with BMI measurement from first trimester (<14 weeks’ gestation), with low BMI (n=59), normal BMI (n=172), and high BMI (n=74), following WHO Asian BMI categories.[32]

[d]Significant for trend. Included only those women with first weight measurement in first trimester (<14 weeks’ gestation, n=306). Excluded from multivariable regression for “maternal risk factors” due to limitation of sample size.

[e]Excluded from multivariable regression analysis for “maternal risk factors” due to collinearity with status (refugee or migrant).

[f]Total number of observations included in multivariable regression for “infant risk factors” for stunting and underweight (n=381). Adjusted for length of gestation, infant sex and infant age.

[g]Excluded from multivariable regression analysis for “infant risk factors” due to collinearity with small-for-gestational age.

[h]A total of 9 data points missing (n=381).
