# Supplementary material for: Feeding practices and risk factors for chronic infant undernutrition among refugees and migrants along the Thailand-Myanmar border: a mixed-methods study
Source: BMC Public Health. 2019 Nov 28;19:1586. doi: 10.1186/s12889-019-7825-7 (PMC6883662; doi:10.1186/s12889-019-7825-7)
Supplement: Supplementary file 3 — Additional file 3: Table S2. Additional quotes from focus group discussions. [file 12889_2019_7825_MOESM3_ESM.pdf]

Table S2. Summary of themes and representative quotes from FGD participants from Mae La (MLA), Mawker Thai (MKT), and Wang Pha (WPA).

Themes	Quote	Site	Ethnicity/parity
Attitudes (experiential and instrumental)			
Infant malnutrition	"My neighbor's child became sick and then became malnourished, losing a lot of weight. Her body became very thin so that the joints in her arms and legs became prominent (visible).<br>I saw one child who had a big belly and you could see every rib; the arms and legs were thin and the baby could not walk—he could only lay on the bed."	MLA	Karen/multiparous
	"Sometimes the parents don't feed their baby enough food[…]. If the baby receives enough food then they get energy and if it is not enough then the baby can't grow up well. […] Malnutrition is a kind of energy. Children can't grow up because the food that they eat does not turn into energy."	MKT	Karen/multiparous
Infant feeding practices	"Yes. Even if the baby drinks breastmilk, sometimes their mouth becomes dry and we have to give a few drops of water. May be sometimes they might be thirsty like an adult. We think like that. We give just a few drops of water not much, only one to two drops."	MKT	Karen/multiparous
	"To 'cool' the baby's stomach because the baby has colic [mother's perception that the baby is hungry]. We do to help the baby get healthy and strong. I don't give much food, just one or two spoons. From 3 months until 6 months, I give only rice. After 6 months I give other foods."	MKT	Burman/multiparous
	"I think it is ok to feed potato but not meat and fish because the baby is too young and I'm afraid the baby might get worms. If the baby has worms then he/she might have stomach pain or discomfort."	MKT	Burman/nulliparous
	"Of course we should feed meat also. We should give balance diet to the baby. It is OK to begin feeding meat at one year or more than one year."<br>"We can boil beef and stir-fry it for the baby also. Maybe I will begin feeding meat at 8 or 9 months when the baby grows teeth."	MKT	Burman/nulliparous

Themes	Quote	Site	Ethnicity/parity
	"I will start feeding egg yolk at 6 months."<br>"At 4 or 5 months, we'll feed rice powder, then by 8 or 9 months, we can mix with some meat or other food. Before 8 months the baby will not have teeth and when we feed only soft rice it is easy for them to consume."<br>"I don't have experiences so I just assume that feeding meat at or after one year is OK."		
	"I will begin feeding foods like meat and fish at one year of age."<br>"I think the baby can eat fish at the age of 10 months; I saw some mothers add fish and feed it to their baby."<br>"I think it is ok to feed potato but not meat and fish because the baby is too young and I'm afraid the baby might get worms. If the baby has worms then he/she might have stomach pain or discomfort."	MKT	Burman/nulliparous
	"I'll start introducing other foods like fish and eggs at one year of age or depending on the baby. If the baby is healthy and can eat any other food then I'll start to give at 10 months."<br>"We give the food twice a day, three soup spoonfuls. We give this way until one year. 6 months to one year we give three spoons, and 6 months to 8 months we also give three spoons."<br>"I give once a day, 5 teaspoons at one time. I give like this from 6 months until 1 year."<br>"I think that it is not enough food. For me, I would increase every month. I did not learn from anywhere; I think that if the baby is getting older that their stomach is becoming bigger. I give a different kind of a spoon, but I start feeding with 3 soup spoons then increase monthly and start feeding twice a day."	WPA	Burman/nulliparous
Maternal nutrition	"When the mother eats the energy goes to the baby inside the belly. If the mother does not eat well, the baby will not have good nutrition."	MLA	Muslim
	"The pregnant women who become malnourished may affect the baby who will not have energy. It may be easy for the baby to become ill.	MKT	Burman/multiparous

Themes	Quote	Site	Ethnicity/parity
	If you don't eat, you may have a premature delivery or your baby will have low birthweight."		
	"If we eat too much and become too fat then our uterus might contract and it becomes more difficult to give birth. If you are too fat, you can't give birth when the uterus is contracted, you have to deliver through cesarean section delivery."	MKT	Karen/multiparous
	"[Overweight may] cause a difficult delivery. The baby is too big so it is not easy to come out." "If we eat too much and become too fat then our uterus might contract and it becomes more difficult to give birth. If you are too fat, you can't give birth when the uterus is contracted, you have to deliver through cesarean section delivery."	MKT	Karen/multiparous
Normative influence and motivation to comply			
Infant feeding practices	"Some staff tell us not to feed water. They asked us to try to feed breast milk as much as we can so that the baby will have some energy and strength. However, I still feed water because I'm worried that the baby might be thirsty. I learned this myself, I feed by myself, no one taught me."	MKT	Karen/multiparous
	"I heard from tharamu ["teacher/midwife/doctor"] that breastmilk helps good brain development and bone growth. Some people have money and feed their babies powder milk, but when they grow up their brain is different—they don't develop properly. The tharamu taught me this and I have also seen this with my own eyes. My youngest sister fed her child powder milk and her child developed differently than my child."	MLA	Karen/multiparous
	"The meat may be raw and smell bad. I heard from older women that you can't give the woman who has just delivered fresh meat or fish because it may be harmful. Here, the fresh meat can give the baby's stomach problems. But for us we give sometimes but only after we grill and then re-cook. I don't give meat to my child very often, but my younger sister gave everything and the child had many episodes of diarrhea. She	MLA	Karen/multiparous

Themes	Quote	Site	Ethnicity/parity
	made different foods for her child like fish, chicken but not beef and pork because it's too hard for the child. But the baby could take only a little bit. For my child I didn't give food I only gave breastmilk and some snacks before one year. I waited until he could take hard food by himself before I fed these foods to him. Sometimes I tried to give earlier before one year, but he didn't like it. He began eating meat only after one year of age."		
Personal agency: perceived control and self-efficacy			
Infant malnutrition	"I have two malnourished babies; both were born early at 7 months. I feed breastmilk but the baby did not look strong. The baby has been very thin since was born until now. I feed breastmilk but still the baby did not gain weight. I think this is because of many kinds of problems I face [poverty]. That's why I buy medicine or vitamin for the baby but still, he is malnourished. Both the mother and the baby don't have good nutrition; my baby is now 7 years old and still very thin and not growing."	MLA	Muslim
Infant feeding practices	"We heard from tharamu that we have to start at 6 months, but I have to work so I begin feeding early at 4 months. I also believe that if I feed early, the child will become strong."	MLA	Karen/multiparous
	"Different mothers do differently. For me, when I started food, I will boil meat. Some of the babies don't like meat; if they don't like meat, have to make with eggs or potatoes."<br>"Like you said, my baby does not like meat, I give but they don't like. Now until 4 years they don't like meat. I try to feed egg, morning glory, potatoes, beans, banana, fruit, apple, orange, watermelon—these they like. Meat they don't like. Fish they don't eat."	MLA	Muslim
	"For my baby I start to feed at one year. I prepared fried rice and my baby eats it until he/she feels full. I only feed my baby with nutritious bread between 6 months and one year. I didn't feed rice. I only feed nutritious foods like banana, milk, etc. I feed only food that he/she can eat. If he/she can eat rice then I	WPA	Burman/multiparous

Themes	Quote	Site	Ethnicity/parity
	feed rice, if only bread then I feed bread. But I feed rice when my baby is one year. I never feed my baby the same way as the other mothers feed. Some mothers even feed their baby with hard rice when the baby is only 6-7 months. For me when my baby is 6-7 months, I have not fed my baby with rice yet. I feed only after my baby is one year old. For my baby, the nutritious foods I are breast milk—it is cool and good for the baby so I only breast feed. In addition to breastmilk, I may feed nutritious foods that my baby can eat like bread so that my baby will also has energy. Sometimes I also feed soft drinks and multivitamins to my baby. I may feed banana, when my baby can eat apple, mangoes then I feed it and if my baby can eat some salad then I feed also. I cannot remember exactly which month I can start these types of foods, it is not like that. It depends on the period when the baby can eat or cannot eat yet. I feed anything that the baby can eat.”		
	“My mother used to do it like that so we learn and feed like that. For my baby I start to feed at one year. I prepared fried rice and my baby eats it until he/she feels full. I only feed my baby with nutritious bread between 6 months and one year. I didn’t feed rice. I only feed nutritious foods like banana, milk, etc. I feed only food that he/she can eat. If he/she can eat rice then I feed rice, if only bread then I feed bread. But I feed rice when my baby is one year. I never feed my baby the same way as the other mothers feed. Some mothers even feed their baby with hard rice when the baby is only 6-7 months. For me when my baby is 6-7 months, I have not fed my baby with rice yet. I feed only after my baby is one year old. For my baby, the nutritious foods I are breast milk—it is cool and good for the baby so I only breast feed. In addition to breastmilk, I may feed nutritious foods that my baby can eat like bread so that my baby will also has energy. Sometimes I also feed soft drinks and multivitamins to my baby. I may feed banana, when my baby can eat apple, mangoes then I feed it and if my baby can	WPA	Burman/multiparous

Themes	Quote	Site	Ethnicity/parity
	eat some salad then I feed also. I cannot remember exactly which month I can start these types of foods, it is not like that. It depends on the period when the baby can eat or cannot eat yet. I feed anything that the baby can eat."		
Maternal nutrition	"Now we are pregnant women and when we are pregnant we have poor appetite. Because they cannot eat, some of the women get sick and feel weak. The blood pressure [BP] can get low. When the BP is low then the baby inside my belly is also not strong, will not grow healthy. When it is time for delivery, my baby and I were not strong. This time in this pregnancy, I know I have to take care of my health. During my last pregnancy, I had anemia so the baby also got anemia. Even if they eat, some of the women still have fetuses that do not get energy, do not grow healthy. The mother who has low BP, cannot use her energy like a healthy woman, this fetus will be malnourished. Now, as pregnant women who are older—more than 30 or 40 years—we cannot eat well like when we were young. If we do not eat well, we cannot make good breastmilk. Now, if I deliver this baby I will not have enough breastmilk because of my age so my baby will also not be that strong. Some of the women buy powder milk and give to the baby because of this. Maybe I have enough breastmilk, but anemia is also bad for my breastmilk and for my baby. For anemia, I will have to eat fruit and other nutritious food. Because I am not so poor, I can buy food, but even if I eat, I have low sugar and still have poor nutrition."	MLA	Muslim
